# Editorial: Impaired receptivity of thin endometrium: the mechanism, hormone intervention and strategies

**DOI:** 10.3389/fendo.2024.1432284

**Published:** 2024-05-31

**Authors:** Jing Shu, Xinmei Liu, Rong Li

**Affiliations:** ^1^ Center for Reproductive Medicine, The First Affiliated Hospital, Zhejiang University School of Medicine, Hangzhou, China; ^2^ Obstetrics and Gynecology Hospital, Institute of Reproduction and Development, Fudan University, Shanghai, China; ^3^ Center for Reproductive Medicine, Department of Obstetrics and Gynecology, Peking University Third Hospital, Beijing, China

**Keywords:** thin endometrium, endometrial receptivity, endometrial compaction, autologous platelet-rich plasma therapy, intrauterine adhesion

The journey towards understanding and effectively treating thin endometrium and intrauterine adhesion is a crucial frontier in reproductive medicine. These conditions significantly impact fertility, often resulting in distressing outcomes such as reduced pregnancy rates and recurrent pregnancy losses. The collection of articles in this Research Topic provides comprehensive insights into innovative disease mechanisms, diagnostic advancements, and therapeutic strategies, reflecting significant strides in enhancing reproductive outcomes for affected women.

## Disease mechanisms and diagnostic innovations

Huang et al. highlighted the use of radiomics to optimize endometrial receptivity evaluations, showcasing the potential of imaging techniques to provide deeper insights into the endometrium in patients with unexplained recurrent pregnancy losses. Chen et al.’s meta-analysis involving 16,164 embryo transfer cycles showed generally no differences of endometrial compaction on reproductive outcomes. However, a subgroup analysis based on endometrial compaction rates (ECR), defined as the rate of change in endometrium thickness between the day of progesterone administration and the day of embryo transfer, found that ECR ≥ 15% observed via transvaginal ultrasound was correlated with higher ongoing pregnancy rates, a finding that enhances predictive capabilities in clinical practice.

## Recent advances in treatment

Wang et al. provided a summary of recent advances in the medical, surgical, regenerative, and alternative therapies for thin endometrium, reflecting a multi-modal approach to addressing this challenging condition. Several hormonal agents including estrogen, tamoxifen, growth hormone, human chorionic gonadotropin, and gonadotropin-releasing hormone agonist were discussed in their review. Notably, Ji et al. reported dramatic improvements in endometrial thickness and pregnancy rates following tamoxifen treatment in patients with thin endometrium, underscoring the potential of repurposed medications in enhancing reproductive outcomes. Hormone replacement therapy after hysteroscopic adhesiolysis, combined with periodic balloon dilation, resulted in comparable reproductive outcomes after embryo transfer in Chen et al.’s studies of 234 cases of intrauterine adhesion. The meta-analysis conducted by Tang et al. enrolled 730 patients from 10 clinical studies and concluded that autologous platelet-rich plasma therapy enhanced both the thickness and quality of the endometrium, while Saad-Naguib et al. focused on the recent advances in the potential of mesenchymal stem cells for treating impaired receptivity of thin endometrium. These studies illustrated the growing interest in and viability of complementary and alternative medical practices within the field of reproductive health.

## Complexity and personalization in treatment

This Research Topic not only advances our understanding of thin endometrium and intrauterine adhesion but also challenges us to integrate various scientific disciplines to improve clinical outcomes. The diverse methodologies and findings presented across these articles underscore the complexity of managing thin endometrium and adhesion issues. Despite limitations such as sample size and quality of evidence, these studies raise critical questions about patient-specific clinical characteristics that dictate the suitability of certain treatments and the potential benefits of combined therapies.

This inquiry highlights the pressing need for personalized treatment strategies that consider individual etiological factors and responsiveness to treatment. Such personalized approaches are essential for moving beyond one-size-fits-all solutions, enabling clinicians to offer more tailored and effective interventions.

## Author contributions

JS: Writing – original draft, Writing – review & editing. XL: Writing – review & editing. RL: Writing – review & editing.

